# ﻿*Caridinastellata*, a new species of atyid shrimp (Decapoda, Caridea, Atyidae) with the male description of *Caridinacavernicola* Liang & Zhou, 1993 from Guangxi, China

**DOI:** 10.3897/zookeys.1104.81836

**Published:** 2022-06-14

**Authors:** Guo-Cai Guo, Qing-Hua Chen, Wen-Jian Chen, Chao-Huang Cai, Zhao-Liang Guo

**Affiliations:** 1 Department of animal science, School of Life Science and Engineering, Foshan University (FU), Nanhai 528231, Foshan, Guangdong province, China Foshan University Foshan China; 2 South China institute of environmental sciences, Ministry of ecology and environment, Guangzhou 510520, Guangdong province, China Ministry of ecology and environment Guangzhou China; 3 College of marine sciences, South China agricultural university, Guangzhou 510642, China South China agricultural university Guangzhou China; 4 Hunan Yuanling animal husbandry and fishery affairs center, No. 13, Jinshui Road, Yuanling Town, Yuanling County 419600, Hunan province, China Hunan Yuanling animal husbandry and fishery affairs center Yuanling China

**Keywords:** COI and 16S rRNA, ecology, habitat, levels of threat, new species, south-western China

## Abstract

*Caridinastellata***sp. nov.** is described from streams in Guangxi, south-western China. The new species clearly belongs to “*Caridinaserrata* group” of the genus and shows a morphological similarity with *C.cantonensis* Yu, 1938, *C.serrata* Stimpson, 1860 and *C.pacbo*Do et al. 2020. *Caridinastellata* is distinguished from congeners, based on differences in its male first pleopod and appendix masculina morphology, along with COI and 16S rRNA molecular evidence. The first pleopod endopod in male is rectangle, about 0.70 × length of exopod, about 3.7–3.9 × as long as proximally wide, inner margin concave, bearing nearly equal spine setae, outer margin bearing nearly equal long and dense spine setae; appendix interna well developed, arising from distal 1/5 of endopod, reaching to end of endopod, with cincinuli distally. The new species displays a unique and brightly coloured pattern and, therefore, can be easily recognised in the field. Liang & Zhou, 1993 described *C.cavernicola* from the Lenggu Cave, Du’an County, Guangxi. However, the description was based exclusively on two females. We have collected specimens of both sexes near the type locality and describe herein the previously unknown male and present morphological data on females. Data on the habitat, ecology and levels of threat of the two species are provided and suggest that they should be categorised as vulnerable (VU) under the current IUCN Criteria.

## ﻿Introduction

*Caridina* H. Milne Edwards, 1837, the largest genus of the family Atyidae, contains more than 300 species and the Indo-West Pacific Region is where the highest diversity is centred (De Grave and Fransen 2011; De Grave et al. 2015; Do VT et al. 2020; De Mazancourt et al. 2021). China harbours more than 100 *Caridina* species (over one-third of the total number of known species), with areas of high species richness in Hunan, Yunan, Guizhou, Guangdong, Taiwan and Guangxi Zhuang Autonomous Region (Liang 2004; Guo and Wang 2005; Liu et al. 2006; Li and Li 2010; Cai 2014; Klotz and von Rintelen 2014; Cai and Ng 2018; Chen et al. 2020; Xu et al. 2020; Feng et al. 2021; Zhou et al. 2021).

Guangxi Zhuang Autonomous Region has 89500 km^2^ of karst landscape, accounting for 37.8% of the total area (Liu et al. 2008). The humid subtropical climate, the diverse karst habitats and the enclosed underground environment provide the conditions for all sorts of organisms. The first records of freshwater atyid shrimps from Guangxi go back to Shen (1948), who listed two species *Caridinaelongata* (=*Neocaridinapalmata*Shen (1948)) and *C.hofendopoda* (=*N.hofendopoda*Shen (1948)). Since then, only a handful of publications have dealt with the atyids of Guangxi, to date, only four genera and about 22 species known from the region (Liang and Yan 1981; 1983; Liang and Zhou 1993; Liang 2004; Cai and Ng 2018). This may be due to insufficient sampling in the karst areas, especially underground habitats.

A faunal survey for freshwater shrimps from the karst habitats of Guangxi in 2018–2019 yielded numerous specimens referable to the genus *Caridina.* In comparing these specimens, we found that they do not fit the descriptions of any of the currently identified congeneric species and we hereby recognise them as belonging to a new species, *C.stellata* sp. nov.

*Caridinacavernicola* was described by Liang and Zhou (1993), based on two female specimens collected from a limestone cave in Lenggu Cave, Du’an Yao Autonomous County, Guangxi. Liang (2004), in his monograph of the family Atyidae of China, made an important re-description of *C.cavernicola* with an illustration of the mouthparts. We have collected samples from three sites inside the Chengjiang National Wetland Park, near the type locality of *C.cavernicola*. The opportunity is thus taken to re-describe and provide new figures on the basis of the new material.

Traditional species descriptions primarily utilised morphological differentiation, illustrations and locality data as diagnostic. The incorporation of morphological and molecular data in species delimitation allows a high level of confidence, crucial for both biodiversity and ecological research. Therefore, the molecular analyses and habitat characterisation of the two species through direct observations are provided. Risk assessments for both are also presented and suggest they both should be categorised as vulnerable (VU) under the current IUCN Criteria.

## ﻿Materials and methods

**Figure 1.**
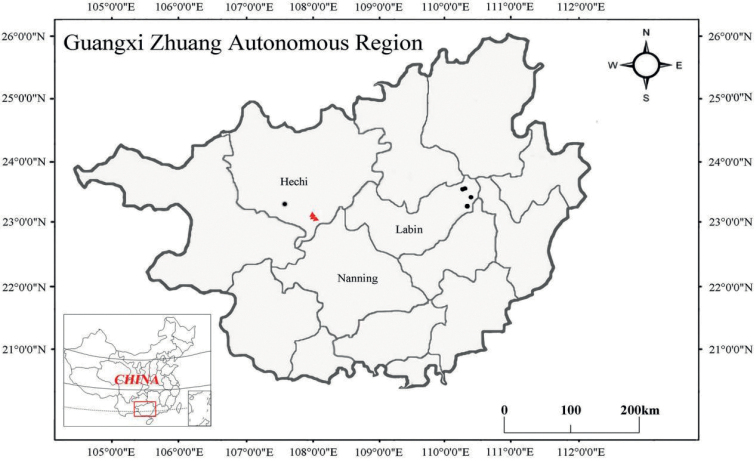
Map indicating rivers in Guangxi Zhuang Autonomous Region, China, with three red triangles showing the sample sites for *Caridinacavernicola* and five black circles showing the sample sites for *Caridinastellata* sp. nov.

**Sample collection.** The shrimp samples were obtained from the karst habitats of Guangxi (Fig. 1). A sturdy long-handled, fine-meshed dip net (mesh size 0.6 mm) was used to collect the shrimp. The sampling scene was recorded with photographs and video-recordings. Specimens were placed in oxygenated polythene bags, anaesthetised with ice and transported back to the hotel. They were later photographed and fixed in 75% ethanol for further morphological examination and molecular analysis. All collection sites were georeferenced using a GPS.

### ﻿Morphological analysis

Specimens were examined using a dissecting microscope (Olympus SZX7). Morphometric measurements on selected characters and illustrations were made using a digital camera (DP22) mounted on a stereomicroscope (Olympus SZX7) with Olympus CellSens Entry v.1.18 software. The measuring method of morphometric characters follows that of von Rintelen and Cai (2009).

The following abbreviations are used throughout the text: alt (altitude), cl (carapace length, measured from the postorbital margin to the posterior margin of the carapace), rl (rostral length, measured from the rostral tip to the postorbital margin) and tl (total length, measured from the rostral tip to the posterior margin of the telson). All measurements are in millimetres.

Voucher specimens were deposited in the collection of the Department of Animal Science, School of Life Science and Engineering, Foshan University (FU).

### ﻿Molecular data collection and analysis

An appropriate amount of shrimp abdominal muscle was taken and put in a 1.5 ml centrifuge tube. DNA was extracted according to the instructions of the EasyPure Genomic DNA Kit (TransGen Biotech, Beijing, China) and then stored in a -20 °C freezer.

Segments of COI and 16S rRNA were amplified by using the primers COI-F-Car and COI-R-Car and 16S-F-Car and 16S-R-Car (von Rintelen et al. 2007). PCRs were conducted in 50 μl volume containing 25 μl 2xEasyTaq PCR SuperMix, 20 μl double distilled H_2_O, 2 μl forward primer, 2 μl reverse primer and 1 μl DNA. The reaction conditions for COI and 16S were: 94 °C for 3 min, 35 cycles of 30 sec at 94 °C, 60 sec at 45 °C (COI) or 50 °C (16S) and 60 sec (16S) or 90 sec (COI) at 72 °C were performed, with a final extension step of 72 °C for 5 min. PCR products were forwardly sequenced using primers with an Applied Biosystems 3730 Analyzer (Applied Biosystems, Foster City, CA, USA).

The DNA sequence of *Caridinastellata* sp. nov. has been deposited in GenBank and 59 sequences have been downloaded from GenBank (Table 1). The sequences were aligned with BioEdit software and similarity was searched using the BLAST tool in NCBI. MAFFT 7.313 was used to compare the studied sequences (Katoh and Standley 2013) and the default values were used for each parameter. Finally, a FASTA format file was derived for subsequent analysis. Inter-group mean distance of the shrimps was calculated using MEGA 7.0, based on COI and 16S rRNA, respectively (Kumar et al. 2016). To obtain the best evolutionary model of sequences for Bayesian Inference (BI) and Maximum Likelihood (ML), the best Bayesian Information Criterion (BIC) evolution model selected by ModelFinder (Kalyaanamoorthy et al. 2017) and the BI and ML phylogenetic trees were constructed using MrBayes 3.2.6 (Ronquist et al. 2012) and IQ-Tree 1.6.12 (Nguyen et al. 2015); the best evolutionary models were TIM2+F+G4 (COI) and TPM3u+F+I+G4 (16S rRNA), respectively.

**Table 1.** Species used in the molecular analysis, with details on sampling locations, GenBank accession numbers (COI, 16S rRNA) (a, Klotz W et al. 2014; b, Chen QH et al. 2020; c, Xu DJ et al. 2020; d, Oliveira, C. M. et al. 2019).

Species	Sampling locality	GenBank accession numbers	
		COI	16S rRNA
*C.stellata* sp. nov.	Jinxiu Guangxi	MZ753496	MZ753799
	Jinxiu Guangxi	MZ753497	MZ753800
*C.* cavernicola	Hechi Guangxi	MZ753498	MZ753801
	Hechi Guangxi	MZ753499	MZ753802
C.venusta	China, Lixi Town, from type loc.	KP168812 ^a^	KP168772 ^a^
	China, Lixi Town, from type loc.	KP168813 ^a^	KP168773 ^a^
*C.* sp.	China, Gao Zhou Shi	KP168790 ^a^	KP168761 ^a^
	China, Gao Zhou Shi	KP168791 ^a^	KP168762 ^a^
*C.nanaoensis*	China	KP168792 ^a^	KP168754 ^a^
	China	–	KP168755 ^a^
*C.breviata*	China, from type loc.	KP168788 ^a^	KP168718 ^a^
	China, from type loc.	KP168789 ^a^	KP168719 ^a^
*C.zhujiangensis*	Dong’ao Island, Zhuhai	MN701603 ^b^	MT446448 ^c^
	Dong’ao Island, Zhuhai	MN701604 ^b^	MT446449 ^c^
*C.trifasciata*	Zhuhai China	KP168795 ^a^	KP168765 ^a^
	Zhuhai China	KP168796 ^a^	KP168766 ^a^
*C.sinanensis*	Sinan Guizhou	MT433963 ^c^	MT434874 ^c^
	Sinan Guizhou	MT433964 ^c^	MT434875 ^c^
*C.serrata*	Dong’ao Island, Zhuhai	MN701595 ^b^	MT446454 ^c^
	Dong’ao Island, Zhuhai	MN701596 ^b^	MT446455 ^c^
*C.mariae*	Nankun Mountain, Huizhou	MN701601 ^b^	MT446456 ^c^
	Nankun Mountain, Huizhou	MN701602 ^b^	MT446457 ^c^
*C.lanceifrons*	Dongfang, Hainan	MN701605 ^b^	MT446450 ^c^
	Dongfang, Hainan	MN701606 ^b^	MT446451 ^c^
*C.huananensis*	Yingde, Qingyuan	MN701607 ^b^	MT446452 ^c^
	Yingde, Qingyuan	MN701608 ^b^	MT446453 ^c^
*C.cantonensis*	Qingyuan, China	KP168802 ^a^	KP168720 ^a^
	Qingyuan, China	KP168803 ^a^	KP168721 ^a^
*N.palmata*	Yangshan, Qingyuan	MN701611 ^b^	–
	Yangshan, Qingyuan	MN701612 ^b^	–
	China	–	KP168779 ^a^
	Hong Kong, China	–	KP168780 ^a^
*A.scabra*	Bocas del Toro, Panama	EF489985 ^d^	JF810980 ^d^
	Bocas del Toro, Panama	EF489986 ^d^	JF810981 ^d^

For the ABGD test, we used COI alignment from the phylogenetic analysis, including the outgroup. ABGD was run online (http://wwwabi.snv.jussieu.fr/public/abgd/abgdweb.html) with the following settings: Pmin = 0.001, Pmax = 0.1, Steps = 10; X = 1.0; Nb bins = 20 and implemented models: Kimura (K80) TS/TV (2.0).

## ﻿Results

### ﻿Taxonomy

**Systematic accounts**

**Family Atyidae De Haan, 1849**

#### Genus *Caridina* H. Milne Edwards, 1837

##### ﻿ Caridina stellata sp. nov.

Taxon classificationAnimaliaDecapodaAtyidae

065D72F3-ECA6-5638-9596-0292E7F5CAC3

http://zoobank.org/61D64E94-8898-44A7-AD99-81FF0950ED83

Figs 2 , 3 , 4A, E

###### Material examined.

***Holotype***: male (FU, 2018-11-05-01), cl 5.4 mm, tl 20.8 mm, rl 2.6 mm, a stream near Liuchacun, Jinxiu Town, Jinxiu Yao Autonomous County, Laibin City, Guangxi Zhuang Autonomous Region, China (24°3'59.63"N, 110°17'43.94"E, alt. 622 m), 5 November 2018. ***Paratype***: male (FU,2018-11-05-02), cl 5.3 mm, ***Paratypes***: 15 males (FU, 2018-11-04-03), cl 5.0–6.2 mm; ***Paratypes***: 29 females (FU, 2018-11-05-04), cl 4.9–6.6 mm, same collection data as for holotype.

***Paratypes***: 17 males (FU,2019-03-20-01), cl 4.7–6.8 mm, three females (FU, 2019-03-20-02), cl 4.5–7.4 mm, a stream near Daxincun, Jinxiu Yao Autonomous County, Laibin City, Guangxi Zhuang Autonomous Region, China (23°57'52.77"N, 110°15'10.91"E, alt. 741 m), 20 March 2019.

***Paratypes***: Four males (FU, 2019-03-19-01), cl 4.7–6.8 mm, 34 females, one ovigerous (FU, 2019-03-19-02), cl 4.5–7.4 mm, a stream near Jiajiangcun, Jinxiu Yao Autonomous County, Laibin City, Guangxi Zhuang Autonomous Region, China (24°11'13.18"N, 110°8'36.79"E, alt. 839 m). 19 March 2019.

***Paratypes***: 31 males (FU, 2019-03-19-03), cl 4.7–6.8 mm, 12 females (FU, 2019-03-19-04), cl 4.5–7.4 mm, a stream near Liupai, Jinxiu Yao Autonomous County, Laibin City, Guangxi Zhuang Autonomous Region, China (24°12'12.76"N, 110°8'25.78"E, alt. 510 m), 19 March 2019.

***Paratypes***: 10 males (FU, 2018-11-26-01), cl 4.7–6.8 mm, seven females (FU, 2018-11-26-02), cl 4.5–7.4 mm, a stream of Lotus Hill Scenic Spot, Dahua Yao Autonomous County, Hechi City, Guangxi Zhuang Autonomous Region, China (24°3'8.12"N, 107°38'30.5"E, alt. 350 m), 26 November 2018.

###### Comparative material.

*Caridinacantonensis*: 10 females (cl: 4.8–6.9 mm), eight males (cl: 5.5–6.5 mm), Zaomushan, Foshan City, Guangdong Province (22°44'22"N, 112°46'36"E, alt. 56 m), 17 May 2018.

*Caridinaserrata*: 17 females (CL: 3.3–6.7 mm), three ovigerous females (CL: 3.9–5.7 mm), 17 males (CL: 2.8–5.3 mm), Dong’ao Village, Dong’ao Island, Zhuhai City, Guangdong Province (22°01'12"N, 113°42'26"E, alt. 8.4 m), 23 August 2014.

###### Diagnosis.

Rostrum long, straight, slightly sloping downwards, reaching to end of 2^nd^ segment of antennular peduncle, occasionally reaching to end of 3^rd^ segment of antennular peduncle; rostral formula 6-8+7-16/6-13. 1^st^ pereiopod carpus 0.43–0.71 × as long as chela, 1.2–1.4 × as long as high; chela 1.8–2.4 × as long as broad; fingers 0.80–1.1 × as long as palm. 2^nd^ pereiopodcarpus 1.1–1.3 × as long as chela, 4.0–4.8 × as long as high; chela 2.1–2.4 × as long as broad; fingers 1.1–1.4 × as long as palm. 3^rd^ pereiopod propodus 4.0–5.5 × as long as dactylus, with two rows thin spines on the posterior margin, ischium with one spine on the posterior margin. 5^th^ pereiopod propodus 4.2–5.3 × as long as dactylus, with two rows of thin spines on the posterior and lateral margins, dactylus terminating in one claw, with 35–40 spinules on flexor margin. Endopod of male 1^st^ pleopod extending to 0.68 × exopod length, wider proximally, rectangle, about 3.7–3.9 × as long as wide, appendix interna well developed, arising from distal 1/6 of endopod, reaching end of endopod. Appendix masculina of male 2^nd^ pleopod cylindrical, reaching to 0.58 length of endopod, appendix interna reaching to 0.50 length of appendix masculina. Uropodal diaeresis with 17–19 movable spinules. Eggs 0.84–0.89 × 1.27–1.39 mm in diameter.

###### Description.

***Body***: slender and sub-cylindrical, males up to 30.7 mm tl, females up to 32.5 mm tl.

**Figure 2.**
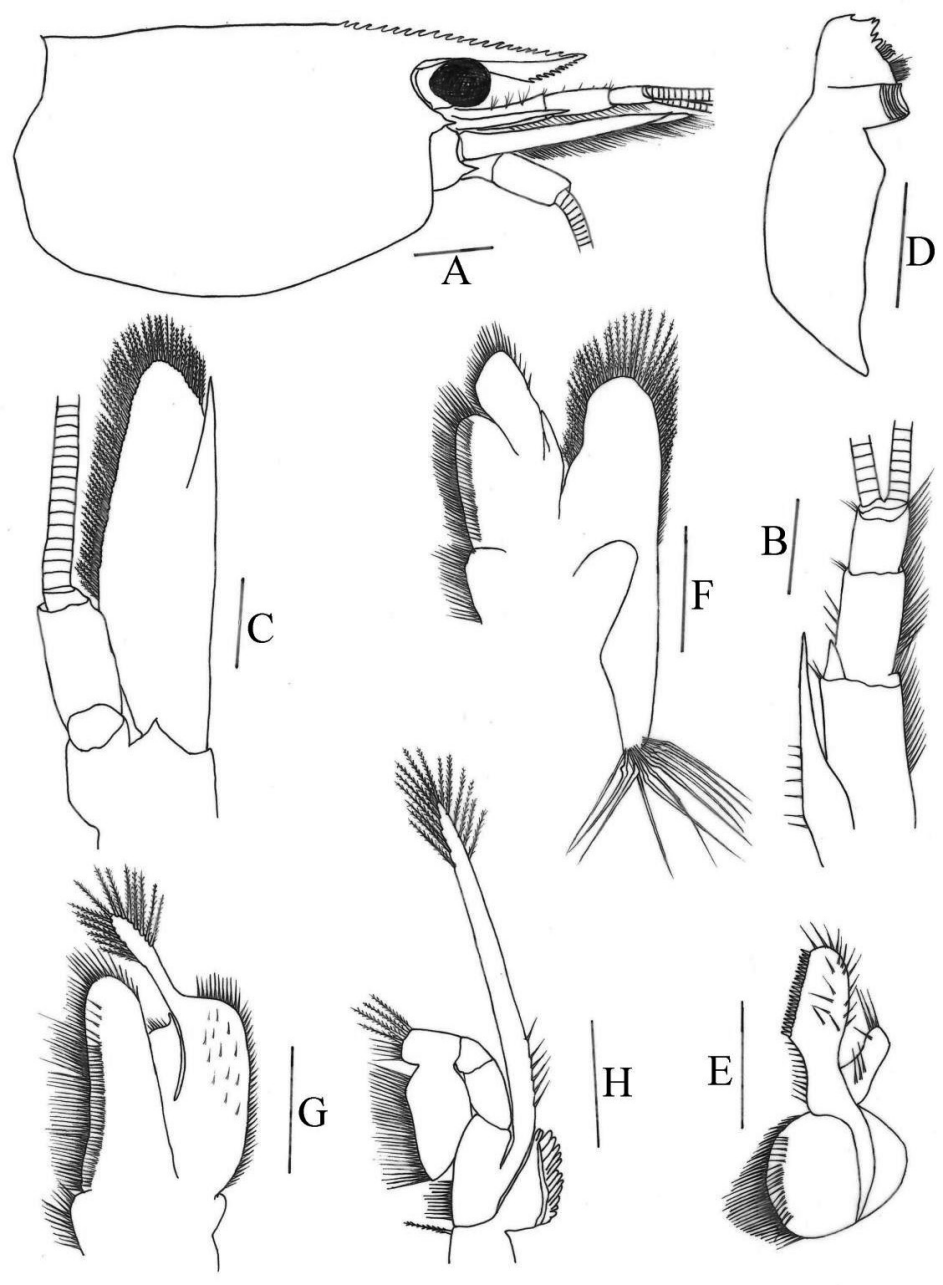
*Caridinastellata* sp. nov. **A** carapace and cephalic appendages, lateral view **B** antennule **C** antenna **D** mandible **E** maxillula **F** maxilla **G** first maxilliped **H** second maxilliped. Scale bars: 1.0 mm (**A**); 0.5 mm (**B–C**); 0.2 mm (**D–H**).

***Rostrum*** (Fig. 2A): Long, straight, slightly sloping downwards, reaching to end of 2^nd^ segment of antennular peduncle, occasionally reaching to end of 3^rd^ segment of antennular peduncle; 0.39–0.48 of cl; armed dorsally with 13–24 teeth, including 6–9 on carapace posterior to orbital margin, ventrally with 6–13 teeth; rostrum formula 6-9+7-15/6-13; lateral carina dividing rostrum into two unequal parts, continuing posteriorly to orbital margin.

***Eyes*** (Fig. 2A): Well developed, on short ocular peduncle, cornea globular.

***Carapace*** (Fig. 2A): Smooth, glabrous; antennal spine acute, fused with inferior orbital angle; pterygostomian margin rectangular, pterygostomian spine absent.

***Antennule*** (Fig. 2B): Peduncle reaching slightly short of scaphocerite; stylocerite long, reaching 0.40 of 2^nd^ segment; anterolateral angle reaching 0.40 of 2^nd^ segment; basal segment as long as combined length of 2^nd^ and 3^rd^ segments, 2^nd^ segment about 0.60 of 1^st^ segment, about 1.6 of 3^rd^ segments; all segments with marginal plumose setae.

***Antenna*** (Fig. 2C): Peduncle about 0.40 × as long as scaphocerite; scaphocerite about 3.5 × as long as wide, outer margin straight, asetose, ending in a strong sub-apical spine, inner and anterior margins with long plumose setae.

***Mandible*** (Fig. 2D): Without palp; left incisor process with five sharp teeth; two groups of setae medially; molar process ridged.

***Maxillula*** (Fig. 2E): Lower lacinia broadly rounded, with several rows of plumose setae; upper lacinia elongate, medial edge straight, with 20–26 strong spinules and simple setae; palp simple, slightly expanded distally, with seven long simple setae.

***Maxilla*** (Fig. 2F): Scaphognathite tapers posteriorly, distally with regular row of long plumose setae and short marginal plumose setae continuing down proximal triangular process, furnished with numerous long plumose setae; upper and middle endite with marginal simple, denticulate and submarginal simple setae, distally with plumose setae; lower endite with long simple marginal setae; palp slightly shorter than the cleft of upper endite, wider proximally than distally, setose.

***First maxilliped*** (Fig. 2G): Palp broadly triangular ending in fringe-like tip and with terminal plumose setae; caridean lobe broad, with marginal plumose setae; exopodal flagellum well developed, with distally marginal plumose setae; ultimate and penultimate segments of endopod indistinctly divided; medial and distal margins of ultimate segment with marginal and sub-marginal rows of simple, denticulate and plumose setae; penultimate segments with marginal long plumose setae.

***Second maxilliped*** (Fig. 2H): Ultimate and penultimate segments of endopod indistinctly divided, reflected against basal segment; inner margin of ultimate, penultimate and basal segments with long setae of various types; exopod flagellum long, slender with marginal plumose setae distally.

Branchial formula typical for genus.

**Figure 3.**
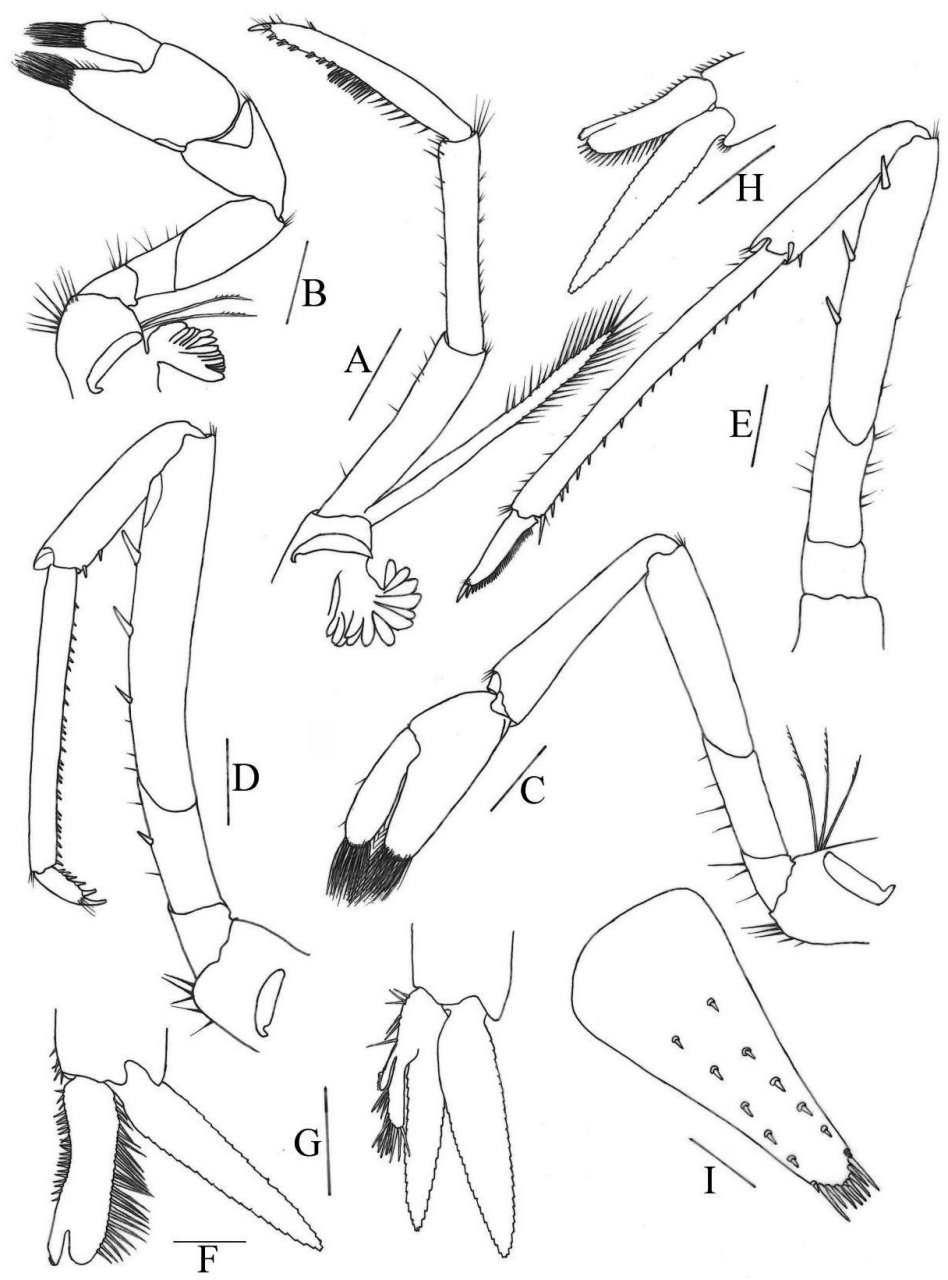
*Caridinastellata* sp. nov. **A** third maxilliped **B** first pereiopod **C** second pereiopod **D** third pereiopod **E** fifth pereiopod **F, H** first male pleopod **G** second male pleopod **I** telson. Scale bars: 0.5 mm (**A–E, I**); 0.2 mm (**F–H**).

***Third maxilliped*** (Fig. 3A): Reaches middle of 3^rd^ antennular peduncle segment, endopod three-segmented, penultimate segment as long as basal segment; distal segment 1.1 × as long as penultimate segment, ending in a large claw-like spine surrounded by simple setae, preceded by 12 spines with double arrangement along distal third of posterior margin, a clump of long and short simple, serrate setae proximally; exopod reaches to end of basal segment of endopod, distal margin with long plumose setae.

***First pereiopod*** (Fig. 3B): Reaches about end of eye; chela 1.8–2.4 × as long as high; 1.4–2.3 × length of carpus; movable finger 2.4–2.8 × as long as wide, 0.80–1.1 × length of palm, setal brushes well developed; carpus excavated disto-dorsally, 1.2–1.4 × as long as wide, 0.85–1.0 × length of merus.

***Second pereiopod*** (Fig. 3C): Reaches about end of 2^nd^ antennular peduncle segment, more slender and longer than first pereiopod; chela 2.1–2.4 × as long as high; 0.76–0.94 × length of carpus; movable finger 3.4–4.9 × as long as wide and 1.1–1.4 × as long as palm, setal brushes well developed; carpus 4.0–4.8 × as long as wide, slightly excavated distally, about 1.1 × length of merus.

***Third pereiopod*** (Fig. 3D): Reaches beyond end of scaphocerite; dactylus 2.0–2.9 × as long as wide, ending in prominent claw-like spine surrounded by simple setae, behind which are 4–5 spines; propodus 4.0–5.5 × length of dactylus, bearing two rows of thin spinules on posterior and lateral margin, 8.0–9.9 × as long as wide; carpus 0.57–0.70 × length of propodus; merus 1.7–2.4 × length of carpus, with about 3–4 strong spines on the posterior margin; ischium with a spine on the posterior margin.

***Fifth pereiopod*** (Fig. 3E): Reaches middle of 2^nd^ segment of antennular peduncle; dactylus 1.7–3.0 × as long as wide, ending in prominent claw-like spine surrounded by simple setae, behind which is a comb-like row of 35–40 spines; propodus 4.2–5.3 × length of dactylus, bearing two rows of spinules on posterior and lateral margins, 9.1–13.0 × as long as wide; carpus 0.43–0.58 × length of propodus; merus 1.4–1.5 × length of carpus, with about 3–4 strong spines on the posterior margin.

First four pereiopods with epipod.

***First pleopod*** (Figs 3F and H): Endopod in male is rectangle, about 0.70 × length of exopod, about 3.7–3.9 × as long as proximally wide, tip rounded, inner margin concave, bearing nearly equal spine setae, outer margin bearing nearly equal long and dense spine setae, distally absent (Fig. 3F) or bearing a few sparse thin spine setae (Fig. 3H); appendix interna well developed, arising from distal 1/5 of endopod, reaching to end of endopod, with cincinuli distally.

***Second pleopod*** (Fig. 3G): Appendix masculina rod-shaped, reaching about 0.60 × length of exopod, inner margin bearing and tip bearing nearly equally long and stout spine setae; appendix interna well developed, reaching about 0.50 × length of appendix masculina, with many cincinuli distally.

***Telson*** (Fig. 3I): 0.42–0.55 × length of cl, distinctly longer than sixth abdominal segment, tapering posteriorly, with a projection, dorsal surface with six pairs of stout movable spine setae including the pair at posterolateral angles; posterior margin with four pairs of intermedial plumose setae, the outer one usually strongest and longest. Exopodite of the uropod bears a series of 17–19 movable spinules along the diaresis.

***Eggs*** 0.84–0.89 × 1.27–1.39 mm in diameter.

###### Colouration.

**Figure 4.**
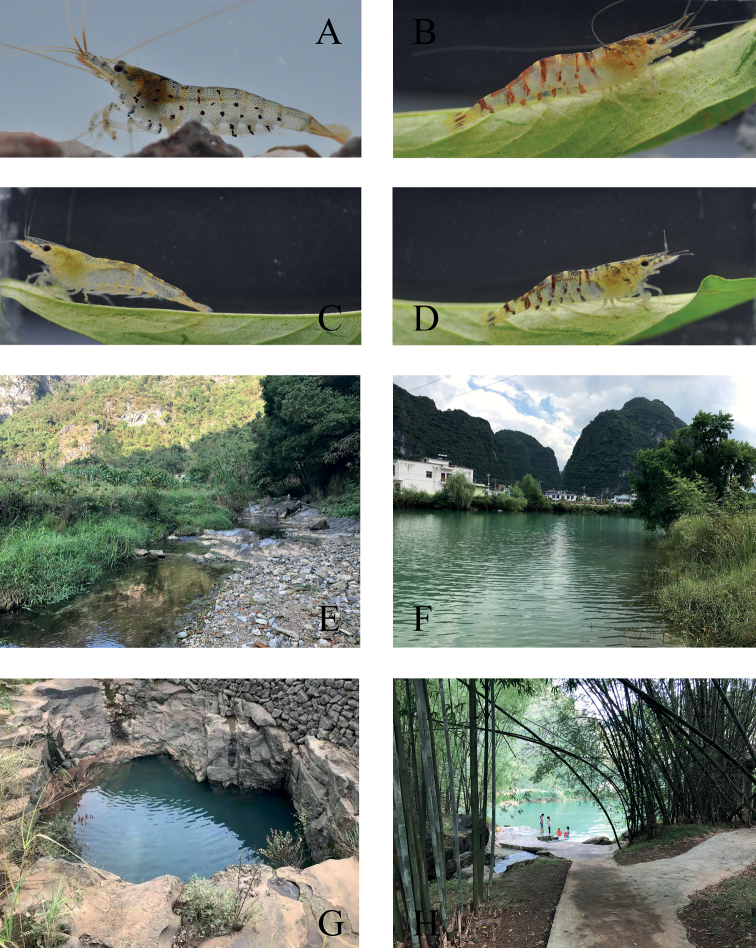
Habitats and live colouration of *Caridinastellata* sp. nov. and *C.cavernicola***A***C.stellata* sp. nov. **B–D***C.cavernicola*; **E–H** surrounding environment of *C.stellata* sp. nov (**E**) and *C.cavernicola* (**F–H**).

Body semi-transparent, light reddish-brown colour, with small red pigment spots scattered on whole body, several large red-brown dots on the tergum and the posterior margin of the carapace, red-brown vertical stripes on topside of the 1^st^ and 2^nd^ pleon and lower lateral side of 1^st^, 3^rd^, 4^th^ and 5^th^ pleon and carapace; appendages transparent, with red-brown stripes in the distal part of each segment; telson and tail fan bright red (Fig. 4A)

###### Etymology.

*Caridinastellata* is named after the Latin word stellatus, for dots, alluding to the pigmented pattern of the body.

###### Remarks.

*Caridinastellata* sp. nov. clearly belongs to the “*Caridinaserrata* group” of the genus and shows a strong morphological similarity with *C.cantonensis* Yu, 1936 in shape and indentation of the rostrum. *Caridinastellata* sp. nov. can be distinguished from *C.cantonensis* by the broad palp of the 1^st^ maxilliped with a finger-like tip (versus without a finger-like tip in *C.cantonensis*); rostrum with more ventral teeth (6–13 versus 2–6 in *C.cantonensis*); the stouter carpus of the 1^st^ pereiopod (1.2–1.4 times as long as wide versus 1.5–1.7 in *C.cantonensis*); the slender endopod of the 1^st^ male pleopod, about 3.7–3.9 × as long as wide, wider proximally (versus 2.5–3.0, wider terminally in *C.cantonensis*); completely different shape of the appendix masculina of male 2^nd^ pleopod (Fig. 3G versus fig. 87r in Liang 2004); and relatively larger eggs, size of developed eggs 0.84–0.89 × 1.27–1.39 mm (versus 0.63–0.72 × 0.99–1.09 mm in *C.cantonensis*). In addition, its distinctive colouration and patterns easily separate the two species when observed in the field.

*Caridinastellata* sp. nov. resembles *C.pacbo* Do, von Rintelen & Dang, 2020 in colouration and pattern and also in the long stylocerite. Moreover, the type locality, Cao Bang Province, Vietnam, is close to Guangxi, China. However, the new species can be distinguished from *C.pacbo* by the longer rostrum, reaching end of 2^nd^ segment of antennular peduncle, 0.39–0.48 of cl (versus close to end of 1^st^ segment, 0.25–0.36 of cl in *C.pacbo*), with more ventral teeth (6–13 teeth versus 0–3 in *C.pacbo*); the stouter carpus of the 1^st^ pereiopod (1.2–1.4 times as long as wide versus 1.3–1.7 in *C.pacbo*); the stouter chela of the 2^nd^ pereiopod (2.1–2.4 times as long as wide versus 2.7–3.1 in *C.pacbo*) with carpus as long as the merus (versus longer than merus in *C.pacbo*); and the slender endopod of the 1^st^ male pleopod (3.7–3.9 × as long as wide versus 2.9–3.3 in *C.pacbo*).

*Caridinastellata* sp. nov. also looks similar to *C.multidentata* Stimpson, 1860 in the colouration and pattern of live individuals. *C.stellata* can be easily distinguished from *C.multidentata* by the longer stylocerite, reaching 0.40 of the 2^nd^ segment of the antennular peduncle (versus 0.70 of the 1^st^ segment of antennular peduncle in *C.multidentata*); with straight rostrum (versus with a crest over orbit in *C.multidentata*), more teeth on carapace posterior to orbital margin (6–9 teeth versus 0 in *C.multidentata*); and large eggs (0.84–0.89 × 1.27–1.39 mm versus 0.23–0.28 × 0.38–0.40 mm in *C.multidentata*).

###### Ecological notes.

*Caridinastellata* appears to be a common atyid species in Guangxi. It was found from four streams in the Jinxiu Yao Autonomous County, Laibin City and also found in Dahua Yao Autonomous County, Hechi City. The environment of the streams is very similar. The streams run through land that is covered by secondary forest, with rocks interspersed with patches of gravel at the bottom (Fig. 4E). The width and depth of the streams were 2.0–3.5 m and 0.3–1.0 m, respectively, with waterfalls and rapids present. The shrimps inhabit vegetation amidst running water, under rocks in lentic environments and even in stagnant water, such as shallow pools. *C.stellata* was found at Jinxiu, in co-existence with the atyid, *Neocaridinapalmata* (Shen 1948) and the palaemonid *Macrobrachiumnipponense*. At Dahua, this species was found also living together with *N.palmata*. The majority of the habitats surveyed had a relatively high density of the new species.

###### Distribution.

Known from Guangxi Zhuang Autonomous Region, southwest China.

### ﻿Molecular phylogenetic results

**Figure 5.**
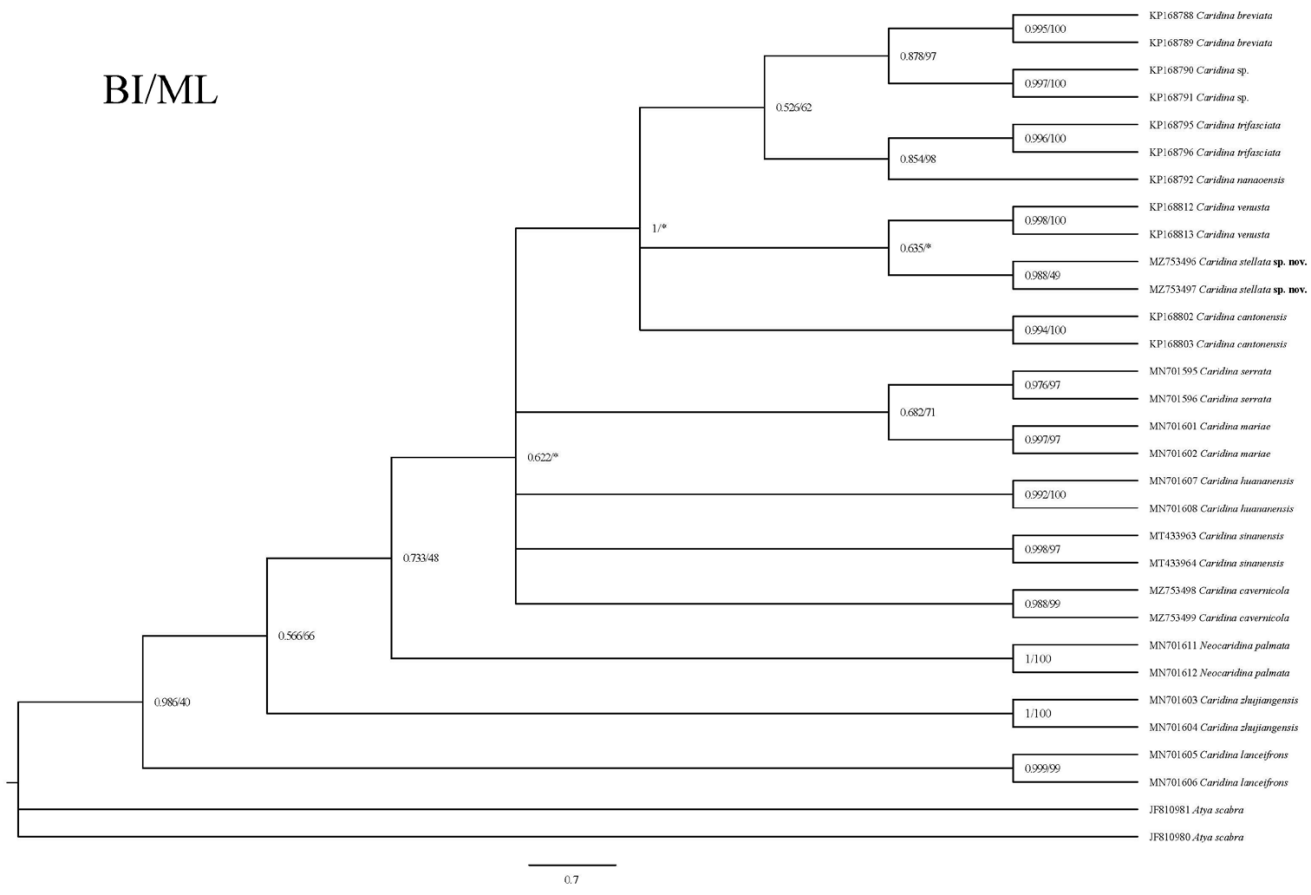
Bayesian Inference (BI) tree and Maximum Likelihood method (ML) tree of 15 atyids and outgroups (*Atyascabra*), based on the COI gene. Support values at the nodes represent posterior probability

**Figure 6.**
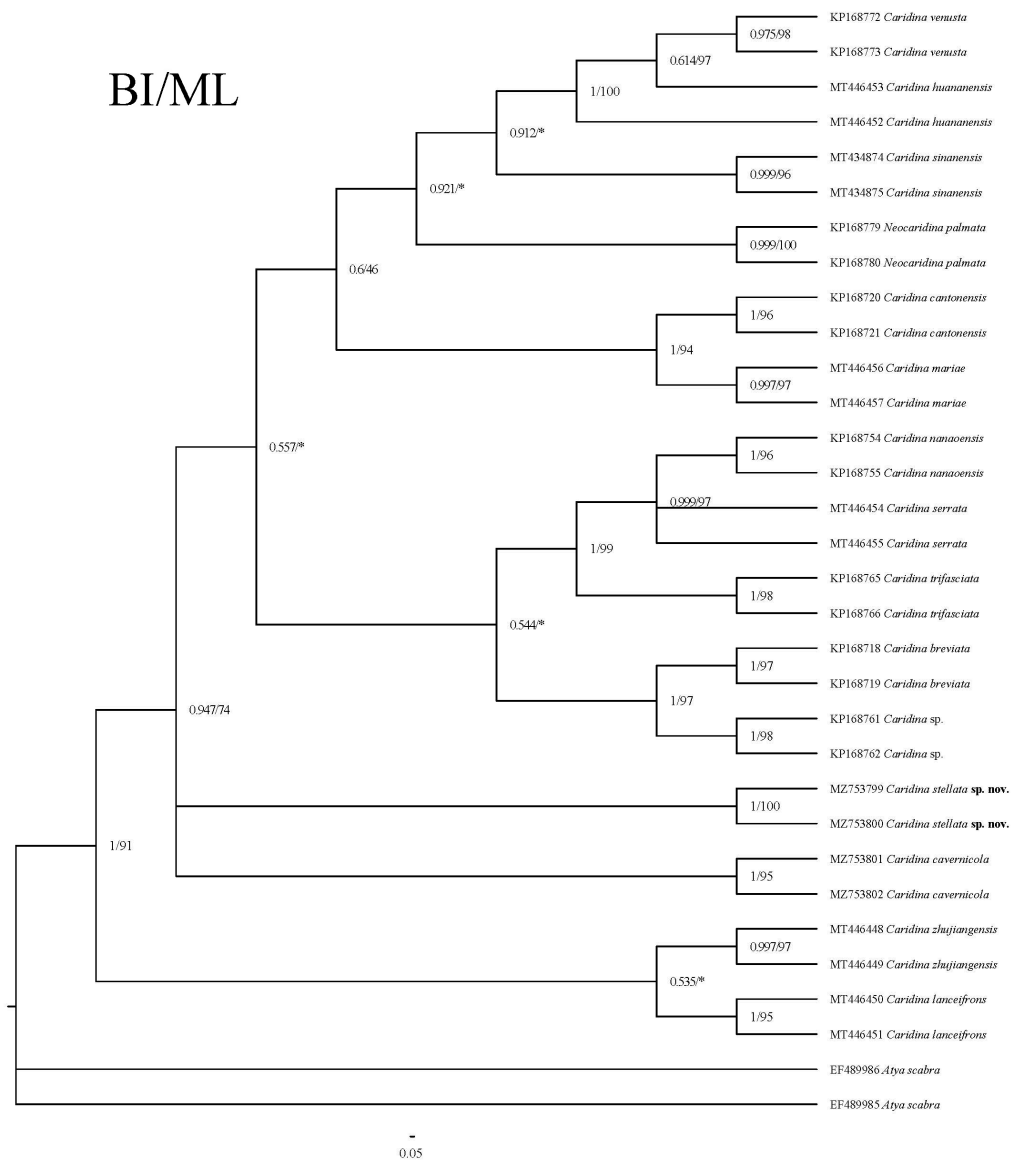
Bayesian Inference (BI) tree and Maximum Likelihood method (ML) tree of 15 atyids and outgroups (*Atyascabra*), based on the 16S rRNA. Support values at the nodes represent posterior probability.

We analysed a total of 31 COI sequences and 32 16S rRNA sequences, 59 of which were from GenBank. The lengths of the sequences are 638 bp (COI) and 461 bp (16S) for the molecular phylogeny analyses. Based on the Kimura Model, inter-group mean distance of 16 species were calculated (Table 2), the genetic distance between *Caridinastellata* sp. nov. and the other nine *Caridina* ranging from 0.126–1.722 (COI) and 0.065–0.112 (16S). Using *Atyascabra* as the outgroup, the Maximum Likelihood phylogenetic tree and Bayesian phylogenetic tree of 16 species of shrimp were constructed (Figs 5, 6). According to the figures, the genetic variability for COI and 16S rRNA are 0.7 and 0.05, respectively. All species can be clustered into a single branch with a relatively high support rate. The new species described above are all well supported and sufficiently distinct from their sister species.

**Table 2.** Pairwise genetic distance amongst 16 species, based on the COI (bottom left) and 16S rRNA (top right) gene.

		1	2	3	4	5	6	7	8	9	10	11	12	13	14	15	16
1	*C.stellata* **sp. nov.**		0.065	0.103	0.098	0.070	0.091	0.080	0.096	0.095	0.095	0.086	0.068	0.112	0.099	0.069	0.236
2	*C.cavernicola*	1.449		0.099	0.095	0.063	0.073	0.077	0.080	0.082	0.077	0.068	0.078	0.106	0.091	0.047	0.213
3	*N.palmata*	1.504	0.203		0.086	0.080	0.094	0.077	0.110	0.086	0.080	0.094	0.076	0.121	0.085	0.096	0.214
4	*C.Venusta*	0.126	1.604	1.770		0.090	0.122	0.088	0.130	0.106	0.070	0.117	0.093	0.133	0.006	0.099	0.235
5	*C.* sp.	0.163	1.578	1.648	0.145		0.068	0.065	0.100	0.065	0.082	0.063	0.075	0.112	0.091	0.026	0.223
6	*C.nanaoensis*	0.158	1.588	1.721	0.139	0.129		0.071	0.109	0.039	0.103	0.004	0.008	0.131	0.123	0.069	0.225
7	*C.cantonensis*	0.144	1.521	1.552	0.134	0.118	0.130		0.086	0.072	0.075	0.071	0.034	0.107	0.086	0.073	0.216
8	*C.zhujiangensis*	1.703	0.244	0.263	1.754	1.850	1.690	1.672		0.109	0.098	0.104	0.101	0.078	0.128	0.099	0.198
9	*C.trifasciata*	0.148	1.578	1.689	0.137	0.144	0.075	0.139	1.745		0.090	0.034	0.075	0.130	0.107	0.069	0.218
10	*C.sinanensis*	1.445	0.143	0.183	1.521	1.525	1.551	1.438	0.240	1.498		0.103	0.070	0.112	0.069	0.081	0.210
11	*C.serrata*	1.460	0.155	0.223	1.575	1.494	1.515	1.402	0.254	1.483	0.155		0.076	0.125	0.118	0.064	0.222
12	*C.mariae*	1.462	0.174	0.244	1.593	1.575	1.547	1.409	0.270	1.497	0.185	0.167		0.129	0.092	0.074	0.229
13	*C.lanceifrons*	1.722	0.268	0.261	1.851	1.742	1.713	1.781	0.239	1.744	0.256	0.261	0.266		0.127	0.115	0.199
14	*C.huananensis*	1.461	0.200	0.251	1.584	1.599	1.580	1.544	0.268	1.594	0.184	0.195	0.210	0.265		0.100	0.230
15	*C.breviata*	0.166	1.574	1.601	0.147	0.059	0.115	0.135	1.851	0.120	1.521	1.475	1.536	1.756	1.583		0.226
16	*A.scabra*	1.823	0.299	0.305	1.785	1.869	1.936	1.970	0.280	1.959	0.278	0.276	0.292	0.266	0.298	1.826	

**Figure 7.**
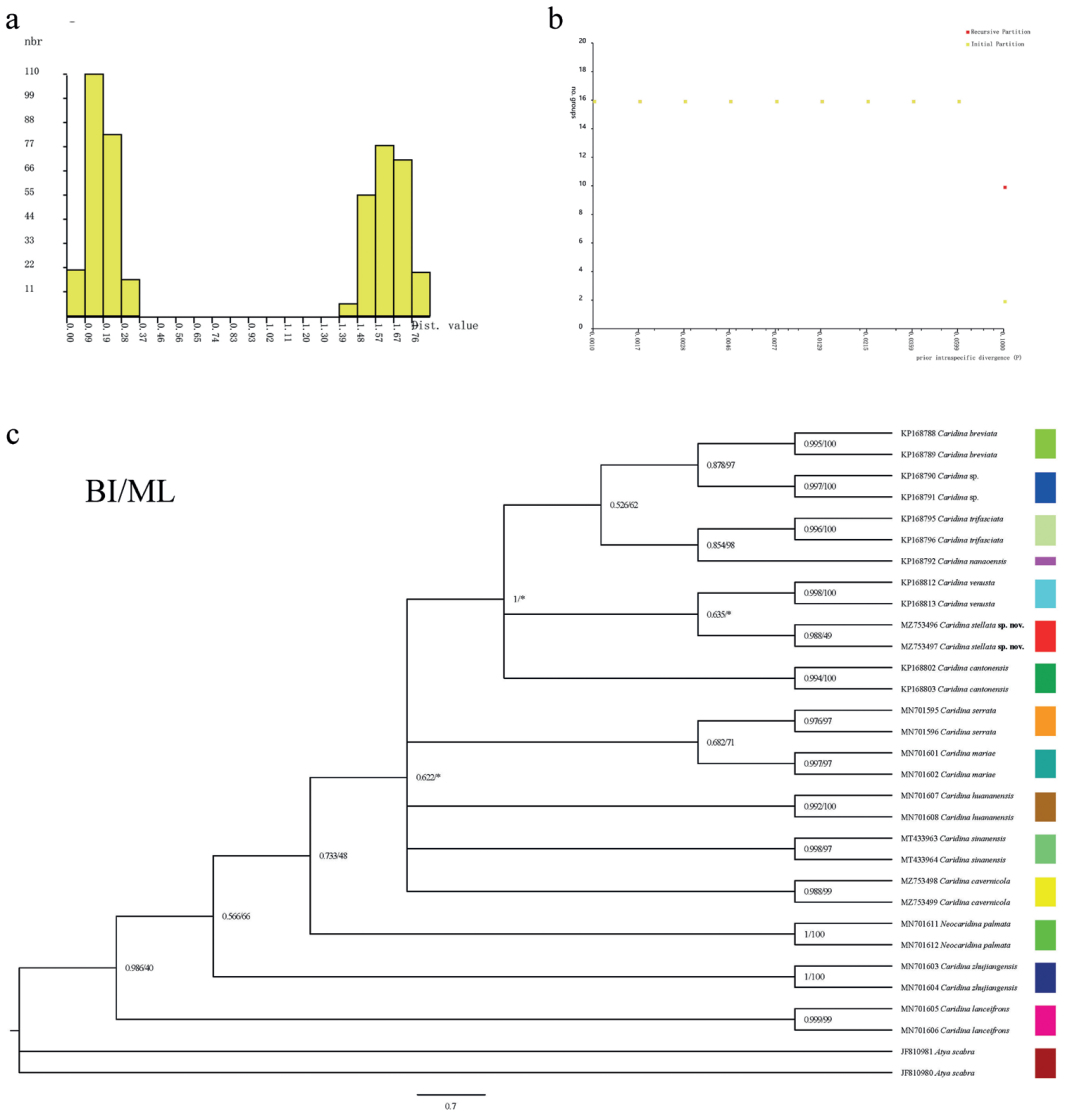
Genetic distance distribution and ABGD partitioning results, based on Kimura (K80) (**a** histogram of distances **b** automatic partition results of ABGD **c** the tree on the left represents the result of the BI/ML analysis and the coloured bars on the right represents the result of model of ABGD test).

In addition, the ABGD division results of 31 COI sequences (including outgroups) in this experiment shows that a significant barcode gap can be formed (Fig. 7a), including both initial division and recursive division. Both recursive and initial divisions were stable (Fig. 7b). When the Prior maximal distances were 0.001000, 0.001668, 0.002783, 0.004642, 0.007743, 0.012915, 0.021544, 0.035938 and 0.059948, they were divided into 16 groups; when the Prior maximal distance was 0.100000, they were divided into 10 groups (Table 3). Therein, the 16 groups were **Group 1**: *C.stellata* sp. nov.; **Group 2**: *C.cavernicola*; **Group 3**: *N.palmata*; **Group 4**: *C.venusta*; **Group 5**: *C.* sp.; **Group 6**: *C.nanaoensis*; **Group 7**: *C.cantonensis*; **Group 8**: *C.trifasciata*; **Group 9**: *C.trifasciata*; **Group 10**: *C.sinanensis*; **Group 11**: *C.serrata*; **Group 12**: *C.mariae*; **Group 13**: *C.lanceifrons*; **Group 14**: *C.huananensis*; **Group 15**: *C.breviata* and **Group 16**: *A.scabra.* This result had a high degree of agreement with the morphological identification results. The partition results of ABGD correspond to the BI/ML tree and the division results of each ABGD were indicated on the BI/ML tree (Fig. 7c).

**Table 3.** The results of the the ABGD division.

Partition	Groups	Prior maximal distance
1	16	0.001000
2	16	0.001668
3	16	0.002783
4	16	0.004642
5	16	0.007743
6	16	0.012915
7	16	0.021544
8	16	0.035938
9	16	0.059948
10	10	0.100000

Combining all of the above results, the results of the division of phylogenetic trees and the classification of species by ABGD are basically the same; the genetic distance supported the molecular-based description of *C.stellata* sp. nov. as a new species.

#### ﻿ Caridina cavernicola

Taxon classificationAnimaliaDecapodaAtyidae

Liang & Zhou, 1993

FDC595F1-54B7-534B-ABAA-F654BB116F74

**Figure 8.**
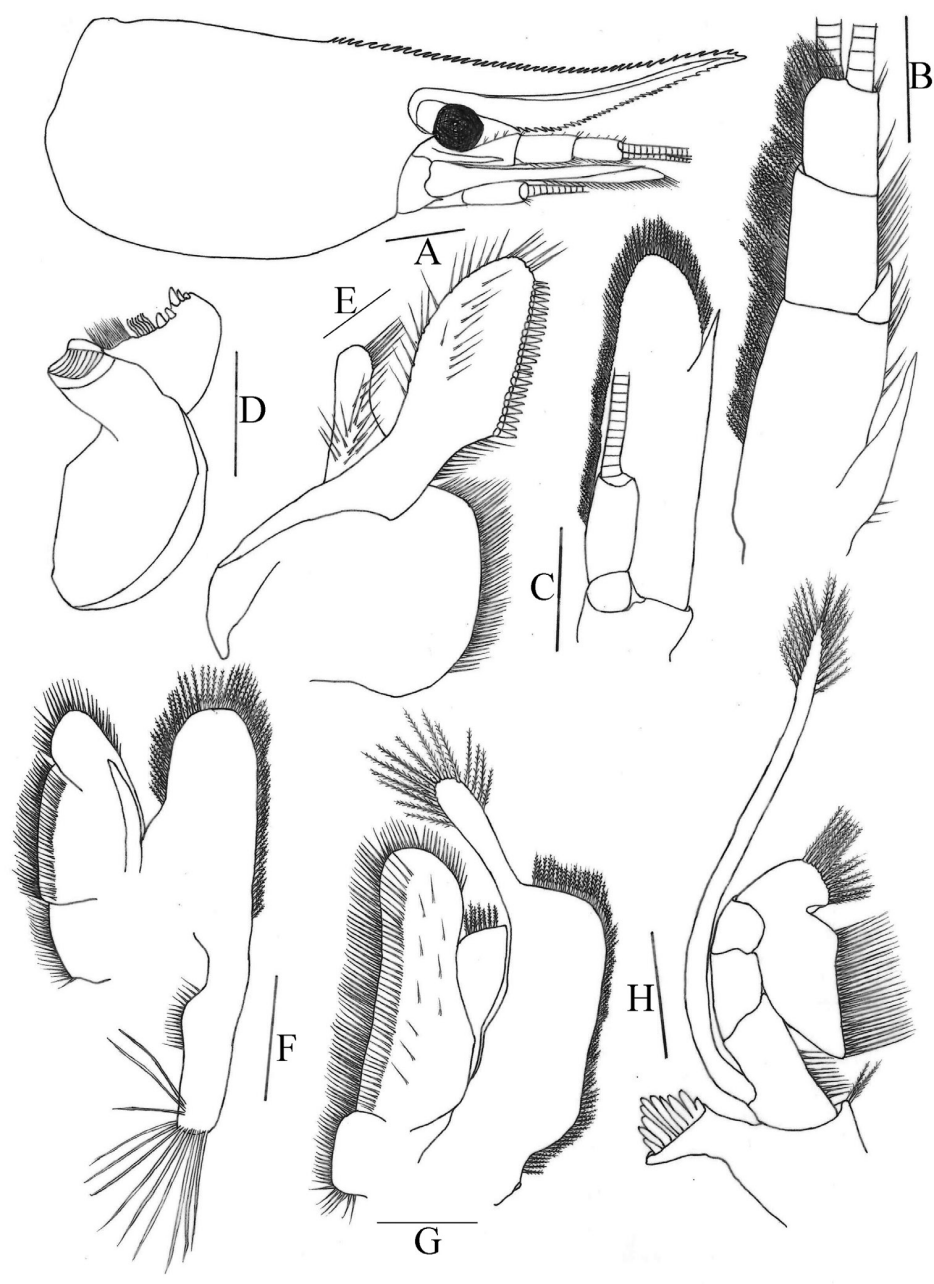
*Caridinacavernicola***A** carapace and cephalic appendages, lateral view **B** antennule **C** antenna **D** mandible **E** maxillula **F** maxilla **G** first maxilliped **H** second maxilliped. Scale bars: 1.0 mm (**A**); 0.5 mm (**B–C**); 0.2 mm (**D–H**).

**Figure 9.**
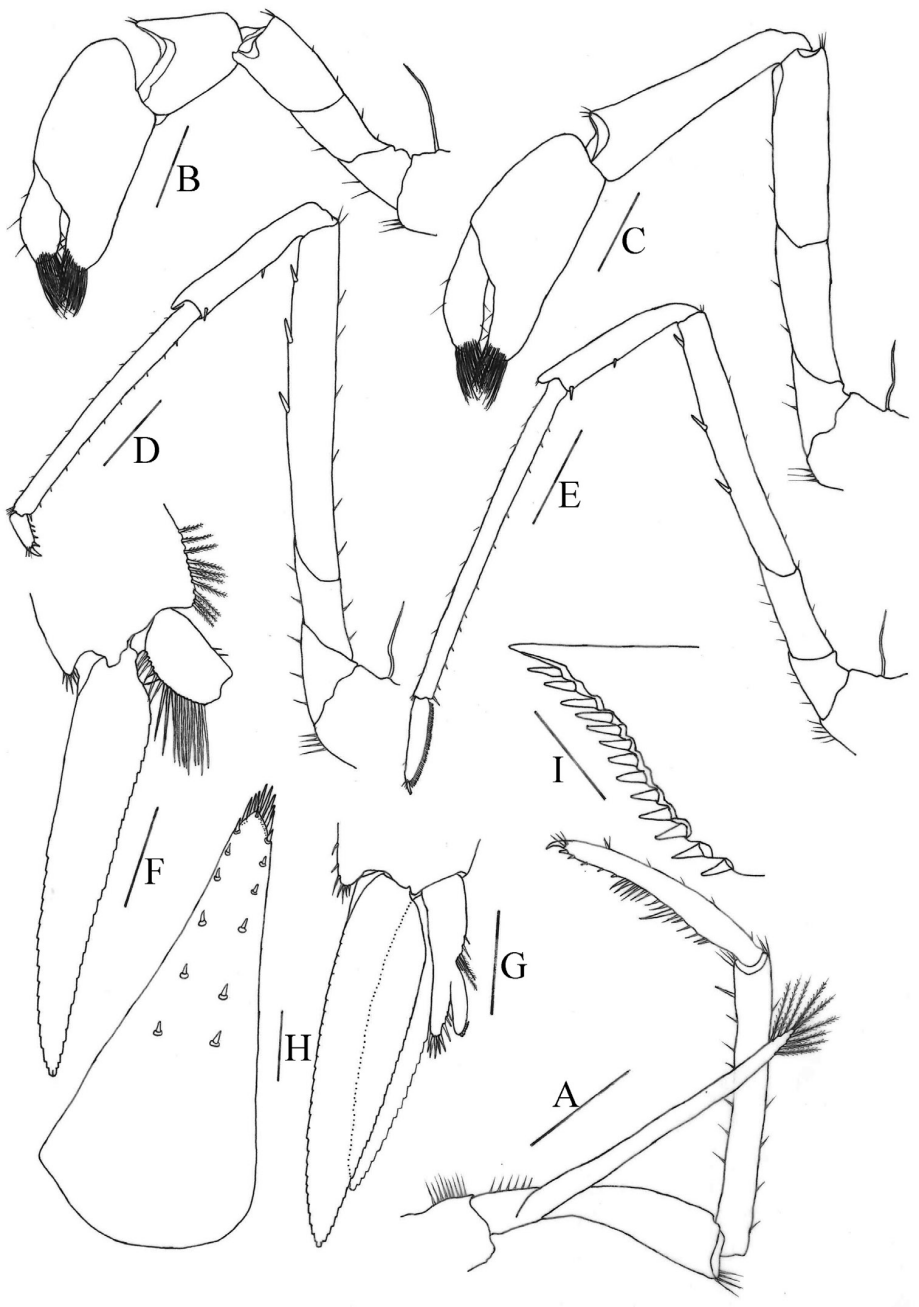
*Caridinacavernicola***A** third maxilliped **B** first pereiopod **C** second pereiopod **D** third pereiopod **E** fourth pereiopod **F** first pleopod **G** second pleopod **H** telson **I** diaeresis of uropodal exopod. Scale bars: 0.5 mm (**A–E, H**); 0.2 mm (**F, G, I**).

Figs 4B–D, F–H , 8 , 9

Caridina cavernicola Liang & Zhou, 1993: 232–234, fig. 2 (1–8). [type locality: Lenggu Cave, Du’an Yao Autonomous County, Guangxi]
Caridina cavernicola Liang 2004: 204–206, fig. 98.

##### Material examined.

Nine males, cl 5.2–7.1 mm, 10 females, cl 5.5–7.8 mm (FU, 2018-11-26-02), Dading Village, Desu Town in the Du’an Chengjiang National Wetland Park (23°56'29"N, 108°0'5"E, alt. 156.17 m), 26 November 2018.3 males, cl 5.0–6.2 mm, five females, cl 5.6–8.1 mm (FU, 2018-11-26-02), near skylight 3, the Du’an Chengjiang National Wetland Park (24°0'11"N, 107°59'13"E, alt. 162.80 m), 26 November 2018. Two males, cl 4.7–5.2 mm, four females, cl 5.2–6.8 mm (FU, 2018-11-26-02), skylight 2, the Du’an Chengjiang National Wetland Park (24°0'24.04"N, 107°59'3.81"E, alt. 150.00 m), 26 November 2018.

##### Description.

Body: slender and sub-cylindrical, males up to 35.3 mm tl, females up to 40.2 mm tl.

***Rostrum*** (Fig. 8A): Long, conspicuously high, tip slightly upturned, beyond one-thirds of rostrum and beyond distal end of scaphocerite; 0.91–1.1 of cl; rostrum formula 7-10+21-33/20-29.

***Carapace*** (Fig. 8A): Smooth, glabrous; antennal spine acute, fused with inferior orbital angle; pterygostomian margin rectangularly rounded, pterygostomian spine absent.

***Antennule*** (Fig. 8B): Peduncle reaching distinctly short of scaphocerite; stylocerite reaching 0.91 of 1^st^ segment; anterolateral angle reaching 0.40–0.5 of 2^nd^ segment; basal segment shorter than combined length of 2^nd^ and 3^rd^ segments, 2^nd^ segment about 0.51 of 1^st^ segment, about 1.2 of 3^rd^ segment; all segments with marginal plumose setae.

***Antenna*** (Fig. 8C): Peduncle about 0.32 × as long as scaphocerite; scaphocerite about 3.2–3.4 × as long as wide, outer margin straight, asetose, ending in a strong sub-apical spine, inner and anterior margins with long plumose setae.

Mouthparts as in figure. ***Mandible*** (Fig. 8D) without palp; left incisor process with five sharp teeth; with two groups of medial setae; molar process ridged. ***Maxillula*** (Fig. 8E) with lower lacinia broadly rounded, with several rows of plumose setae; upper lacinia elongate, medial edge straight, with 23–27 strong spinules and simple setae; palp simple, slightly expanded distally, with numerous long simple setae. ***Maxilla*** (Fig. 8F) with scaphognathite tapering posteriorly, with regular row of long plumose setae distally and short marginal plumose setae continuing down proximal triangular process, furnished with numerous long plumose setae; upper and middle endite with marginal simple, denticulate and submarginal simple setae, with distal plumose setae; lower endite with long simple marginal setae; palp slightly shorter than the cleft of upper endite, wider proximally than distally, setose. ***First maxilliped*** (Fig. 8G) with broad palp and with terminal plumose setae; caridean lobe broad, with marginal plumose setae; exopodal flagellum well developed, with marginal plumose setae distally; ultimate and penultimate segments of endopod indistinctly divided; medial and distal margins of ultimate segment with marginal and sub-marginal rows of simple, denticulate and plumose setae; penultimate segments with marginal long plumose setae. ***Second maxilliped*** (Fig. 8H) with ultimate and penultimate segments of endopod indistinctly divided, reflected against basal segment; inner margin of ultimate, penultimate and basal segments with long setae of various types; exopod flagellum long, slender with marginal plumose setae distally. ***Third maxilliped*** (Fig. 9A) reaches to middle of 3^rd^ antennular peduncle segment, endopod three-segmented, penultimate segment about 1.3 × as long as basal segment; distal segment 0.79 × as long as penultimate segment, ending in a large claw-like spine surrounded by simple setae, preceded by five spines, proximally a clump of long and short simple, serrate setae; exopod long, reaches to half of penultimate segment of endopod, distal margin with long plumose setae. Epipods on first four pereiopods.

***First pereiopod*** (Fig. 9B): Reaches to about end of eye; chela 1.4–2.2 × as long as high; 1.7–1.9 × length of carpus; movable finger 2.4–2.6 × as long as wide, 0.50–0.67 × length of palm, setal brushes well developed; carpus excavated disto-dorsally, 1.3–1.7 × as long as wide, 1.1–1.2 × length of merus.

***Second pereiopod*** (Fig. 9C): Reaches to about end of 2^nd^ antennular peduncle segment, more slender and longer than first pereiopod; chela 2.1–2.3 × as long as high; 0.97 × length of carpus; movable finger 2.9 × as long as wide and 0.91 × as long as palm, setal brushes well developed; carpus 3.3–4.2 × as long as wide, excavated distally, about 1.2 × length of merus.

***Third pereiopod*** (Fig. 9D): Reaches beyond end of scaphocerite; dactylus about 3.3 × as long as wide, ending in prominent claw-like spine surrounded by simple setae, behind which are 4–5 spines; propodus 4.9–6.4 × length of dactylus, bearing a row thin spinules on posterior and lateral margin, about 12.1 × as long as wide; carpus about 0.68 × length of propodus; merus 1.6–1.9 × length of carpus, with about 3 strong spines on the posterior margin.

***Fourth pereiopod*** (Fig. 9E): Reaches middle of 2^nd^ segment of antennular peduncle; dactylus 4.8–5.2 × as long as wide, ending in prominent claw-like spine surrounded by simple setae, behind which is a comb-like row of 61–69 spines; propodus 3.6–4.4 × length of dactylus, bearing a row of spinules on posterior and lateral margins, 16.5–17.1 × as long as wide; carpus 0.50–0.54 × length of propodus; merus 1.3–1.5 × length of carpus, with about three strong spines on the posterior margin.

***First pleopod*** (Fig. 9F): Endopod in male short, rectangle, about 0.26 × length of exopod, about 1.7 × as long as proximally wide, tip concave, inner margin bearing equal two thin spine setae, outer margin bearing nearly equal long and dense spine setae, without an appendix interna.

***Second pleopod*** (Fig. 9G): Endopod about 0.83 × length of exopod; appendix masculina rod-shaped, reaching about 0.49 × length of endopod, inner margin and tip bearing nearly equal spine setae; appendix interna well developed, almost the same size as appendix masculina, reaching about 0.97 × length of appendix masculina, with many cincinuli distally.

***Telson*** (Fig. 9H): 0.44–0.51 × length of cl, distinctly longer than sixth abdominal segment, posterior margin acutely triangular, with a projection, dorsal surface with 6–7 pairs of stout movable spine setae including the pair at posterior lateral angles; posterior margin with four pairs of intermedial plumose setae, lateral pair of spines subequal to intermedian pairs. Exopodite of the uropod bears a series of 12–15 movable spinules along the diaresis.

***Eggs*** 0.80–0.92 × 1.37–1.40 mm in diameter.

##### Colouration.

Body translucent, rust brown, with small red pigment spots scattered on whole body, with a broad red-brown vertical stripe on each abdominal segment; appendages transparent (Figs 4B–D).

##### Remarks.

*Caridinacavernicola* was known from only two females and one juvenile specimen when it was first collected from a limestone cave in Lenggu Cave, Du’an Yao Autonomous County, Hechi City, Guangxi. Only the name of the cave is mentioned without detailed environmental information and body colour of the shrimps (Liang and Zhou 1993). Attempts to find the Lenggu Cave through enquiring the exact location from local government departments and residents was unfruitful. However, it was most fortunate that we have collected samples from three sites inside the Chengjiang National Wetland Park. This species is abundant amongst leaf litter and the fibrous roots of riparian trees and plants along the edges of the Chengjiang River. It was also found in small populations in a skylight. No difference was found between individuals collected from the river and skylight sites. We can speculate that the underground water of the skylight may be connected to the Chengjiang River. This also indicates that the shrimp likely only recently invaded the cave environment and can occupy both epigean and hypogean karst habitats.

Liang (2004) mentioned the status of this species as questionable due to some unusual characters, such as: 1^st^, 2^nd^ pereiopod chela stout; 2^nd^ pereiopod carpus disto-dorsally excavated; 3^rd^, 4^th^, 5^th^ pereiopod propodus posterior margin with numerous long plumose setae. Through this study, however, we have found that the morphological and genetic data are congruent and that this species clearly belongs to the genus *Caridina*.

##### Ecological notes.

Chengjiang National Wetland Park is located in Du’an Yao Autonomous County, Hechi City, Guangxi. It is also a part of the Du’an Subterranean River National Geopark, Guangxi. The Park mainly consists of the Chengjiang River and integrated farming wetland, river wetland and urban wetland, covering a total area of 8.64 km^2^, with a width of 11.7 km and a length of 24.2 km. Chengjiang River originates from two skylights, one is Yantan Pool, located at the foot of Guanyin Mountain in Jiudun Village, Daxing Town, the other is Dongtan Pool, located in Taiyang Village, Daxing Town. Chengjiang River belongs to the Red River system, one of the tributaries of the Pearl River system. The river is 50–80 m wide and 5–10 m deep. Chengjiang River and its associated wetlands are also home to many other rare and endangered endemic species of plants and animals. The seaweed flower, *Otteliaacuminate* is an endangered aquatic plant that is only found in China (Yunnan, Guizhou, Guangxi and Hainan) and can be found in Chengjiang River. Peach blossom jellyfish, *Craspedacusta* sp., appears in skylight 1 at Zhuqing Tun, Dongmiao Village, Dongmiao Township. The teleostean fish, *Metziaformosae* is listed as vulnerable (VU) in the China Red Data of endangered animals: fishes (Yue and Chen 1998) and is also found here. *Yunnaniluspulcherrimus*, *Aphyocyprispulchrilineata*, *Metzialonginasus*, *Silurusduanensis* and *Bibarbabibarba* are endemic species of Du’an County (Ye et al. 2016).

*Caridinacavernicola* were caught alongside *Neocaridinapalmata* (Shen 1948) and *Macrobrachiumnipponense* in river sites.

##### Distribution.

Know from Guangxi Zhuang Autonomous Region, southwest China.

## ﻿Discussion

This research was done by comparing DNA barcode sequences, phylogenetic trees were constructed, genetic distances were calculated and ABGD software was applied to classify species. Research results had found that significant barcode gaps can be formed and Automatic partition results of ABGD grouped *Caridinastellata* sp. nov. into a separate group. The results of the division of phylogenetic trees were basically the same as those of ABGD on species. The present results confirmed that the integrated use of DNA barcoding (BI/ML tree, K2P distance and ABGD) are efficient and reliable methods for delineation and genetic identification of *Caridinastellata* sp. nov. as a new species. At the same time, combined with the research of morphology, this can promote the development of taxonomy.

During the recent sampling along the karst habitats of Du’an County, Guangxi, two species of *Caridina* have been collected. *Caridinacavernicola* was originally found from a subterranean stream near Du’an County, but further surveys have found dense populations in the Chengjiang River.

Narrow distributions, high diversity and a high level of endemism are characteristic of the genus *Caridina*. These isolated and vicariant *Caridina* species occur in karst locations, generally considered as an important part of the natural heritage. They may be particularly vulnerable to anthropogenic activities and face risk of extinction in the future; therefore, more urgent conservation attention may be warranted. Defining potential threats posed by human activities to all *Caridina* species would be the first step in effectively managing their conservation. Guangxi karst landforms have good potential for tourism due to the beautiful natural landscape and ideal climate. *Caridinastellata* sp. nov. is only known from a few hill stream localities. One stream is located in Lotus Hill Scenic Area. The increasing exploitation of tourist resources for human use fails to recognise the needs of the species that live there. Moreover, *C.stellata* has striking colouration and patterns that have received particular attention amongst aquarists. In recent years, it has been collected, reared and traded in commercial aquarium industries. The wild populations will inevitably be threatened by over-harvesting. *Caridinacavernicola* is also facing the same issues due to its distribution in relatively disturbed areas, the Chengjiang National Wetland Park. The population is experiencing considerable stresses and disturbances. The Chengjiang River Basin is surrounded by densely-populated towns. Domestic sewage discharge and wastewater from washing clothes, cleaning vegetables and even people taking showers are problems in many parts of the Chengjiang River (Fig. 4H). Over-exploitation of water for hydroelectricity, agriculture and tourism are also likely to be critical problems. Several hydrotechnical constructions are built along the river, such as irrigation facilities, dams for power generation and landscape. The invasive fish, *Oreochromisniloticus* Linnaeus 1758, can be seen everywhere in the river. In addition, *C.cavernicola* is potentially an ornamental species due to their attractive colour pattern (Figs 4B–D) and may also be impacted by harvesting for the aquarium trade in the future. Given that information, these threats are expected to lead to habitat destruction and fragmentation, resulting in population decline and this situation might be further aggravated by the lack of regulations and active measures for protection. Therefore, the risk of extinction for these two species can be classified into vulnerable (VU) using the IUCN Red List Categories and Criteria (IUCN, 2019, version 4.1).

To deal with the anthropogenic disturbances, regular monitoring of wild population changes should be carried out and campaigns that promote environmental education and raise tourists, awareness of the importance of biodiversity should be encouraged. In addition, developing commercial aquaculture techniques for the captive breeding of ornamental species is urgently needed in order to guarantee a sustainable supply of shrimp for the industry. This can have certain advantages in reducing the risk of extinction if populations can be maintained in captivity in the long term. More thorough sampling efforts, coupled with molecular identification, will be needed in the future to better understand the diversity and distribution of *Caridina* species in Guangxi. The number of described species will doubtlessly increase dramatically in the near future and more information on their evolution and ecology will be known as more karst habitats are studied. The biodiversity conservation of karst habitats will be greatly strengthened.

## Supplementary Material

XML Treatment for Caridina stellata

XML Treatment for Caridina cavernicola
